# In vivo tumor cell adhesion in the pulmonary microvasculature is exclusively mediated by tumor cell - endothelial cell interaction

**DOI:** 10.1186/1471-2407-10-177

**Published:** 2010-04-30

**Authors:** Peter Gassmann, Mi-Li Kang, Soeren T Mees, Joerg Haier

**Affiliations:** 1 Department of General and Visceral Surgery; University Hospital Muenster; Muenster; Germany

## Abstract

**Background:**

Metastasis formation is the leading cause of death among colon cancer patients. We established a new in-situ model of in vivo microscopy of the lung to analyse initiating events of metastatic tumor cell adhesion within this typical metastatic target of colon cancer.

**Methods:**

Anaesthetized CD rats were mechanically ventilated and 10^6 ^human HT-29LMM and T84 colon cancer cells were injected intracardially as single cell suspensions. Quantitative in vivo microscopy of the lung was performed in 10 minute intervals for a total of 40 minutes beginning with the time of injection.

**Results:**

After vehicle treatment of HT-29LMM controls 15.2 ± 5.3; 14.2 ± 7.5; 11.4 ± 5.5; and 15.4 ± 6.5 cells/20 microscopic fields were found adherent within the pulmonary microvasculature in each 10 minute interval. Similar numbers were found after injection of the lung metastasis derived T84 cell line and after treatment of HT-29LMM with unspecific mouse control-IgG. Subsequently, HT-29LMM cells were treated with function blocking antibodies against β1-, β4-, and αv-integrins wich also did not impair tumor cell adhesion in the lung. In contrast, after hydrolization of sialylated glycoproteins on the cells' surface by neuraminidase, we observed impairment of tumor cell adhesion by more than 50% (p < 0.05). The same degree of impairment was achieved by inhibition of P- and L-selectins via animal treatment with fucoidan (p < 0.05) and also by inhibition of the Thomson-Friedenreich (TF)-antigen (p < 0.05).

**Conclusions:**

These results demonstrate that the initial colon cancer cell adhesion in the capillaries of the lung is predominantly mediated by tumor cell - endothelial cell interactions, possibly supported by platelets. In contrast to reports of earlier studies that metastatic tumor cell adhesion occurs through integrin mediated binding of extracellular matrix proteins in liver, in the lung, the continuously lined endothelium appears to be specifically targeted by circulating tumor cells.

## Background

One third of patients diagnosed with colo-rectal cancer will eventually die from this disease representing ~600,000 deaths worldwide per year [1]. Most of these colo-rectal cancer related deaths are due to distant metastatic growth rather than local tumor progression. As in almost any cancer, the pattern of metastatic growth is non-random and in the case of colo-rectal cancer liver and lung are predominant metastatic targets, beside lymph nodes and the peritoneal cavity [2]. The organ specific character of metastatic growth has already been recognised by S. Paget [3] in the late 19^th ^century and his 'seed-and-soil concept' has been modified by additional findings and dissected into single steps, outlined in the literature as the 'metastatic cascade' [4].

Based on various in vivo and ex vivo techniques, several groups suggested mechanically restricted tumor cell embolism in the first capillary bed entered by circulating tumor cells [4,5]. In contrast, our own group [6] and other authors [7] demonstrated specific tumor cell adhesion in the microcirculation of the liver and lung [8,9]. Furthermore, injected human colon cancer cells showed an organ specific pattern of cell adhesion in rats, mimicking the clinical picture of metastatic colon cancer [10]. Glinksii et al. [11] reported organ-selective metastatic tumor cell arrest of human breast cancer cells in a mouse model, following the typical clinical pattern of metastatic breast cancer diseases, and α2- and α6- integrin expression of colon carcinomas was found to correlate with their metastatic potential and patient prognosis [12]

Circulating tumor cells adhere to microvascular endothelial cells (EC) and subendothelial extracellular matrix (ECM) proteins by different sets of adhesion molecules. Subsequent and regulated transendothelial cell migration taking place at secondary sites in response to several microenvironmental factors as has been numerously reported [6-10,13]. Despite the morphologic similarities of leukocyte and tumor cell adhesion, there are distinct differences between inflammatory cells and cancer cells due to the available surface adhesion molecules and intracellular signalling cascades. Obviously, malignant cells originating from a variety of tissues differ with regard to available adhesion molecules for cell - cell or cell - ECM interactions. For example, E-Selectin mediated adhesion to endothelial cells has been established for inflammatory leucocytes as well as several human cancer cells [7-9]

α4β1-integrin binding of endothelial Vascular Cellular Adhesion Molecule-1 (VCAM) was associated with leukocyte adhesion [14] to endothelial cells, as well as adhesion of human melanoma cells [15] to the endothelium, but appears not to be involved in prostate cancer cell adhesion [16]. These findings suggest different adhesion mechanisms of hematogenous cells, non-epithelial and epithelial cancer cells during their arrest within capillaries. Recently, we reported comprehensive data on cell surface molecules involved in initial metastatic tumor cell adhesion of human colon carcinoma cells within the hepatic microvasculature in vivo [17,6]. These results showed preferential arrest of circulating colon carcinoma cells within the liver via integrins [9,10] The aim of the present study was to evaluate the adhesion molecules and mechanisms involved in the organ-specific adhesion of human colon cancer cells in the lung as their second metastatic target organ. To this end we established a new in-situ model for quantitative in vivo fluorescence microscopy of the ventilated and perfused lung.

## Methods

### Materials and Cells

Media (RPMI1640; DME/F12) and fetal bovine serum (FBS) were purchased from GIBCO-BRL (Karlsruhe, Germany). All other chemicals were purchased from Sigma (Deisenhofen, Germany).

Human highly-metastatic HT-29LMM colon carcinoma cells (I. Fidler; Houston, TX) were cultured in RPMI1640 medium and human T84 colon cancer cells (A. Nussrath, Atlanta), derived from a lung metastasis, were cultured in DMEM-F12 medium containing 10% FBS without antibiotics in humidified 5% CO_2_/95% air at 37°C. Confluent cell monolayers were used during the log-phase of growth. Prior to the experiments cells were rinsed with Calcium-Magnesium-free phosphate buffered solution (CMF-PBS), trypsinized and kept in serum-free adhesion medium (RPMI1640 containing bovine serum albumin [BSA] 1%) for 60 min for reconstitution of cell surface proteins. Trypsinized cells were resuspended as single cell suspension in CMF-PBS at a final concentration of 1 × 10^6 ^cells/ml. This preparation did not interfere with adhesive and migrative properties in vitro [18]. For inhibition experiments, various antibodies or neuraminidase-V (C. perfringens) (Sigma) were added during reconstitution of the cells as indicated below.

### Flow cytometry analyses of adhesion molecules

For flowcytometric analysis of adhesion molecules, cells were trypsinized and washed in serum free medium. After reconstitution for 60 min, cells were fixed with fresh 1% paraformaldehyde and processed following a standardized protocol. The following primary antibodies were used for detection of certain cell adhesion molecules: anti-human integrin β1 mAb (clone P4C10, Chemicon, Hofheim, Germany), anti-human integrin β4 mAb (clone ASC-8, Chemicon), anti-human integrin β3 (clone N-20, Santa Cruz,), anti-human αv mAb (clone 272-17E6, Calbiochem, Darmstadt, Germany), anti-human α1 integrin mAb (clone SP2/0, Upstate, Lake Placid NY), anti-human α2 integrin mAb (clone 16B4, Serotec, Oxford, UK), anti-human α3 integrin mAb (clone ASC-1, Chemicon), anti-human α4 integrin mAb (kindly provided by J. Eble; Münster, Germany), anti-human α5 mAb (clone JBS5, Serotec), anti-human α6 mAb (clone 4F10, Serotec), anti-sLe_a _(clone KM93; Chemicon), anti sLe_x _(clone KM231; Chemicon), anti galectin-3 (clone B2C10; Santa Cruz Biotechnology). Negative and isotype controls were similarly processed. Corresponding Alexa-fluor568-labeled secondary antibodies (Molecular Probes, Leiden, Netherlands) were used for flow cytometry analysis. For detection of Thomson-Friedenreich antigen, cell were incubated with fluorescence labeled Lectin from Arachis hypogaea (L0881; Sigma) specifically binding TF-antigen in increasing concentrations. Flow cytometry was done using EPIC XL (Beckman Coulter, Krefeld, Germany).

### Intravital Fluorescence Video Microscopy

**Figure 1:**
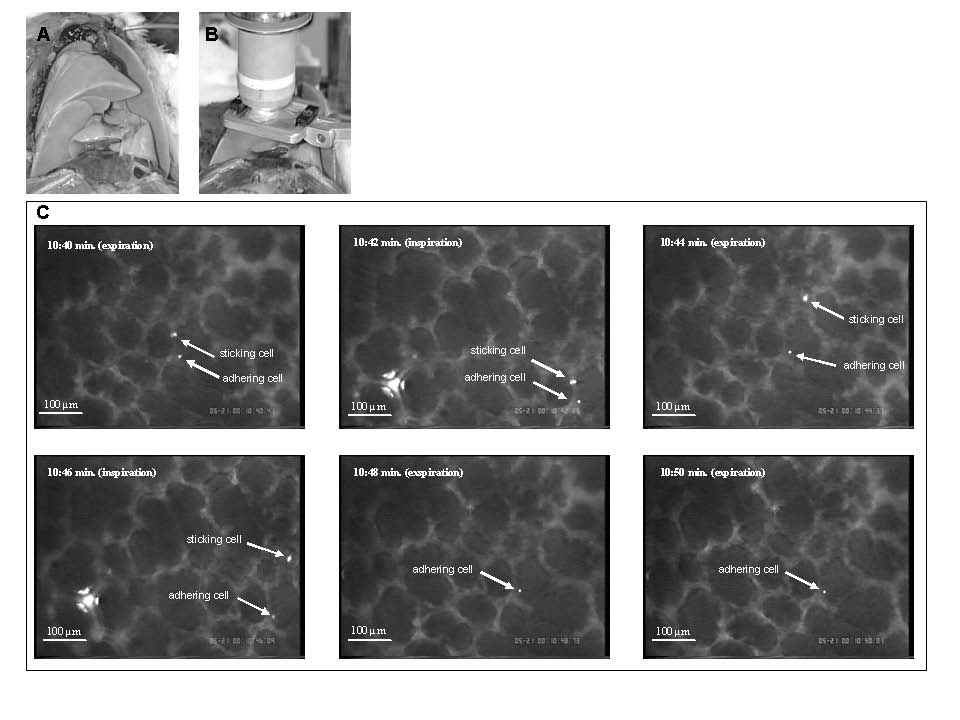
**Set-up for in vivo microscopy of the ventilated rat lung**. **A: **After placement of a catheter central to the heart through the right carotid artery, a tracheostomy and canulation of the trachea was performed for establishing mechanical ventilation. The chest wall was removed and the right lower lobe was exposed. **B: **A glass cover slip, mounted on a specially designed holding device, was placed upon the right lower lobe with minimal pressure to serve as artificial pleura, and the animal was placed under an upright in vivo microscope. ***Time frame (10 sec.) of stably adherent and sticking human colon cancer cell***. **C: ***Full scale microscopic pictures representative for in vivo microscopy of the ventilated and *physiologically perfused lung in-situ. The presented pictures were taken at the indicated time intervals after tumor cell injection. One fluorescence-labeled cell is stably adherent to the vascular wall over the 10 second interval of observation. The other cell ("sticking cell") is adherent for a few seconds to the wall without establishing stable adhesion and finally recirculates through the pulmonary capillaries. For quantitative analysis of tumor cell adhesion, the lung surface was screened for stably adherent cells in a standardized fashion in 10 minute intervals for a total of 40 minutes.

Male Sprague-Dawley rats (CD rats; 250-300 g; Charles River) were cared for in accordance with the standards of the German Council on Animal Care, under an approved protocol of the local Animal Welfare Committee (LANUV NRW 9.93.2.10.36.07.122). Rats were anesthetized using inhalation of isofluorane (Curamed, Karlsruhe, Germany) and N_2_O. A permanent catheter was introduced through the right carotid artery and the tip placed centrally to the heart. An open tracheotomy was performed and the animal was mechanically ventilated through a canula placed and fixed in the trachea by a small animal respirator (Harvard Small Animal Ventilator, Harvard Apparatus, Hugstetten, Germany) at a rate of 35-40/minute and a tidal volume of 3,5 - 4 ml. Adequate ventilation was confirmed by arterial blood gas analysis. The anterior chest wall was carefully removed without disturbances of the lung surface avoiding atelectases. The animal was placed under an upright in vivo fluorescence microscope (Zeiss, Oberkochen; Germany) and fluorescence microscopy was performed through a thin glass cover slip mounted on a specially designed holder, carefully placed upon the lung's surface with minimal pressure. For positive contrast of the blood vessels, the animals were slowly injected with 500 μg fluorescine (FITC) labelled dextran 10,000 kD. Using a similar protocol, earlier studies demonstrated stable hemodynamic conditions and constant microcirculation in the lungs for at least 60 minutes [19]. Microscopy was performed through a water immersion objective with 20× magnification (Figure 1A/B).

### Semiquantitative analysis of tumor cell adhesion in vivo

10^6 ^CalceinAM labeled human HT-29LMM colon carcinoma cells were injected as a single cell suspension in 1 ml CMF-PBS over 1 minute. Standardized lung surface microscopy was begun immediately after the injection was completed. The lung surface was continuously screened in a standardized manner recording the number of single adherent tumor cells per microscopic field in inspiration as described before [10]. Tumor cells adhering for ≥10 seconds were defined as stably adherent. Microscopy was performed in 10 minute intervals for a total of 40 minutes. Emboli of cell clumps were excluded from analysis.

The cell numbers given are the number of adherent cells per 20 microscopic fields for each 10 minute time interval and are expressed as means ± standard deviation from n independent experiments (animals injected).

### Involvement of Cell Adhesion Molecules

For inhibition experiments cells were incubated either with function blocking monoclonal antibodies (mAbs), type V-neuraminidase or conditioned medium. For some experiments the animals were treated with 0.8 mg fucoidane per kg bodyweight 15 minutes prior to cell injection as described by Preobrazhenskaya et al. [20] for inhibition of selectin mediated adhesion.

For integrin and galectin-3 inhibition cells were incubated with the appropriate antibodies (1-3 μg/ml, 60 min) during reconstitution to block specific integrins prior to the assays. The following mAbs were used: anti-β1 integrin (clone P4C10, Chemicon, Hofheim, Germany), anti β4-integrin (clone ASC-8; Chemicon), anti-α_v _(clone 272-17E6, Calbiochem, Darmstadt, Germany), anti-galectin-3 (clone B2C10; Santa Cruz Biotechnology). This pretreatment has been shown to interfere with the regulation of cell adhesion in HT-29 cells in-vitro [21] and in vivo [6,17]. Unspecific mouse IgG (Sigma) was used as control.

Enzymatic hydrolisation of cell surface glycoprotein sialic residues to inhibit Selectin ligands was achieved by incubating cells with 0.01 U/ml type V-neuraminidase (from C. perfringens; Sigma, Deisenhofen; Germany). This treatment has been shown to interfere with tumor cell adhesion in vivo [6]. Vehicle (untreated) cells were used as control. The inhibition of the Thomson-Friedenreich antigen (TF antigen) was realized by, incubating cells with conditioned medium from the supernatant of JAA-F11 hybridoma cells that produce function-blocking mouse IgG against the TF antigen [22,11]. Control cells were incubated with unconditioned medium.

Prior to injection cells were washed and resuspended as single cell suspension in CMF-PBS at a final concentration of 1 × 10^6 ^cells/ml.

### Laser scanning microscopy for ex situ evaluation of specific adhesion

Cells were labeled with CellTracker Green CMFDA fluorescence staining (Molecular Probes) (0.01 mg/ml) for 3D-reconstruction of adherent tumor cells within the pulmonary microcirculation and the animal was injected with Vybrad-Dil-LDL (0.04 mg/animal) for labeling of the endothelium. Both dyes are stable during formalin fixation and paraffin-embedding. 40 minutes after the tumor cell injection in situ fixation was carried out by infusing CMF free PBS at a physiologic pressure of 10-15 mmHg through the inferior vena cava, followed by a 3.75% formalin infusion. The inflated lungs were removed carefully avoiding the creation of atelectases and kept in formalin for three days. 25 μm sections were mounted on glass slides after paraffin embedding. Three dimensional reconstructions of fluorescence-labeled sections were processed using a laser-scanning confocal microscope (Nikon, Düsseldorf; Germany) and the lucia5-software package (Nikon).

## Results

### HT 29 LMM adhere to the pulmonary microvasculature

The first fluorescence labeled cells were seen in the pulmonary microcirculation as soon as 1 minute after the initiation of the tumor cell injection. While some cells passed the pulmonary capillaries without any sign of mechanical size restriction, first adhesive interaction of the injected cells with the pulmonary capillary system were observed within minutes. All adherent cells were localized within the pulmonary capillaries and no adhesive cells were observed within larger pre- or post-capillary vessels. The mean diameter of pulmonary capillaries determined by in vivo microscopy was 14.3 μm ± 2.5 μm. Perfusion of the capillaries was usually maintained despite the presence of adhering cells. Once tumor cells were adherent for at least 10 seconds, significant recirculation was not observed within the observation period. In contrast to earlier observations under comparable conditions regarding the liver [23], some cells were found sticking to the lung vessel walls and eventually recirculating before establishing a firm adhesion as it has been described for leucocytes (Figure 1C). Rolling of cells prior to adhesion was not observed.

**Figure 2:**
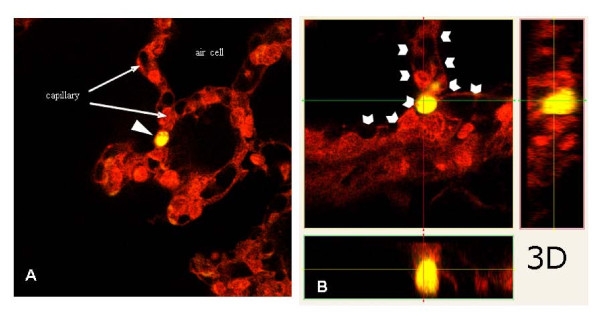
**A/B: Laser scanning microscopy of adherent tumor cell (formalin fixed)**. After in situ perfusion with PBS with physiologic pressure through the inferior V. Cava, lungs were fixated in situ by formalin infused with physiologic pressure. After paraffin embedding, 25 μm sections were analysed by 3D laser scanning microscopy. Three dimensional reconstruction revealed tumor cells (A, yellow cell with white arrow head) adherent to the microvascular endothelium (B, white arrow heads) and leaving a perfused vessel lumen without signs of mechanical size-restricted cell arrest.

Three dimensional reconstructions of the lungs after in situ fixation and confocal laserscanning microscopy revealed specific adhesive interactions between tumor cells and the microvasculature with persistent free capillary lumen (Figure 2). In vivo microscopy of these areas demonstrated remaining perfusion within the capillaries next to the adhering tumor cells (Figure 1C). It is worthwhile to mention that extravasated tumor cells, e.g. cells in the alveolar lumen, were not detected by confocal laser scanning microscopy 40 min after cell injection.

### Adhesive properties of HT-29LMM and T84 colon cancer cells

The human HT-29LMM colon cancer cell line was derived from repeated in vivo passaging in mice [24]. To validate the cell line's adhesive properties within the rodent model used here, we compared the pulmonary tumor cell adhesion of HT-29LMM with the adhesive properties of human T84 colon cancer cells derived from a lung metastasis. After injection of T84 cells treated with unspecific mouse IgG (n = 6) 16.7 ± 9.0; 15.8 ± 5.1; 14.1 ± 5.6; and 13.0 ± 1.9 cells/20 microscopic fields were found adherent in the lung at 10; 20; 30; and 40 minutes after injection, respectively. Similar processing of HT-29LMM cells resulted in comparable numbers of adhesive cells within the pulmonary microcirculation at all time intervals (Table 1). HT-29LMM cells were used for further experiments due to better comparability with results from earlier studies using a similar microscopic technique within the liver.

**Table 1:** Pulmonary tumor cell adhesion in vivo

Treatment	Time intervall after cell injection [min]			
	
	0-10	11-20	21-30	31-40
	**adherent cells/20 microscopic fileds [mean ± SD]**			
**T 84** **control IgG [n = 6]**	16.7 ± 9.0	15.8 ± 5.1	14.1 ± 5.6	13.0 ± 1.9

**HT-29 LMM** **control IgG [n = 9]**	13.5 ± 5.1	13.5 ± 4.8	11.1 ± 2.7	13.5 ± 2.3

**anti integrin-β1 [n = 7]**	11.4 ± 4.7	11.8 ± 4.4	12.9 ± 2.7	12.3 ± 3.8
**anti integrin-β4 [n = 10]**	13.4 ± 7.6	10.7 ± 4.0	10.4 ± 3.2	11.0 ± 4.5
**anti integrin-αv [n = 8]**	10.6 ± 5.1	10.1 ± 4.1	9.5 ± 2.5	11.7 ± 2.7
				
**vehicle [n = 12]**	12.8 ± 4.7	13.2 ± 6.7	10.9 ± 3.9	15.0 ± 5.9

**neuraminidase type V [n = 8]**	7.4 ± 3.0	6.6 ± 2.3*	6.5 ± 2.6*	7.9 ± 2.9**
**fucoidane (animals) [n = 7]**	11.6 ± 6.9	7.4 ± 3.1*	7.2 ± 3.3*	5.6 ± 2.5**
				
**control medium [n = 5]**	10.9 ± 5.1	18.3 ± 10.1	16.4 ± 3.9	14.8 ± 6.1

**anti galectin-3 [n = 5]**	18.8 ± 6.7	13.3 ± 2.4	13.1 ± 2.9	12.3 ± 3.2
**anti TF [n = 4]**	11.5 ± 14.7	7.9 ± 4.7*	8.6 ± 3.7**	7.2 ± 4.0*
**anti TF + anti galectin-3 [n = 5]**	11.6 ± 1.0	7.4 ± 2.8**	7.3 ± 2.3**	6.5 ± 1.4**

ANOVA and post hoc (LSD):

* p < 0.05

** p < 0.001

### Expression of adhesion molecules on human HT 29 LMM colon cancer cells

**Figure 3:**
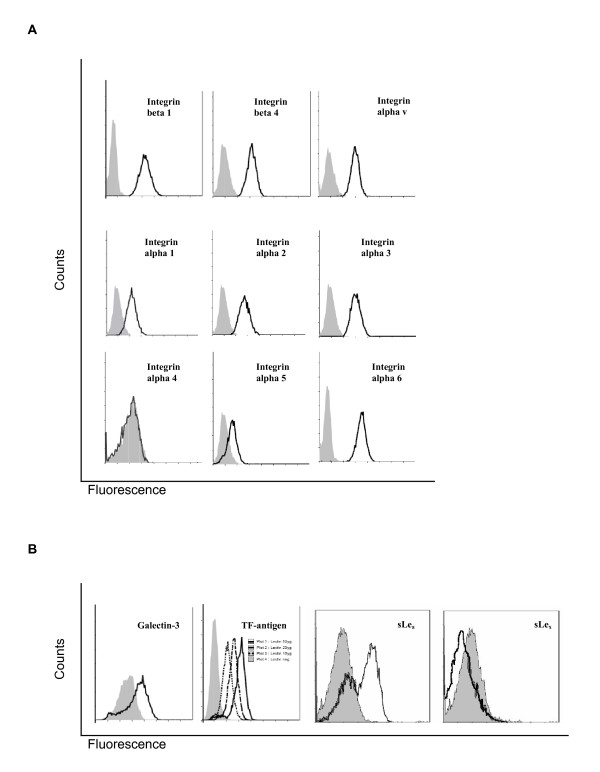
**Expression of adhesion molecules at HT-29LMM surfaces**. ***A Integrin expression: ***Flow-cytometry analysis revealed predominant expression of β_1_-, β_4_-, as well as α_1_-, α_2_-, α_3_- α_6_- and α_v_- integrins on HT-29 LMM cells. Only weak expression of α_5_-integrins; α_4_-integrins are not expressed. ***B Glycoprotein expression: ***E-Selectin binding sLe-a was found on HT-29LMM in the absence of sLe-x. TF-antigen and galectin-3 are both present for endothelial cell binding.

Identification of potentially involved sets of adhesion molecules was performed by flow cytometry analysis defining adhesion molecule expression at the tumor cell surface. Human HT-29LMM colon cancer cells showed significant expression of β1-, β4-, α1-, α2-, α3-, α6-, and αv-integrin subunits. While only weak α5-integrin expression was found, α4-integrins were not detected, on the surface of this cell line of epithelial origin (Figure 3A).

In accordance with other authors [9] we found sialyl Lewis-a (sLe-a), on HT-29LMM cells, while sialyl Lewis-x (sLe-X) was absent from the cells' surface (Figure 3B).

Furthermore, HT-29LMM cells were found to express Galectin-3 and Thomson-Friedenreich antigen (TF) simultaneously (Figure 3B).

### Integrins are not involved in initial colon cancer cell adhesion within the pulmonary microvasculature

We reported earlier that inhibition α6β1- and α6β4-integrins as well as inhibition of αvβ5-integrins significantly interfered with metastatic tumor cell adhesion within the hepatic sinusoids [6,17]. Since adhesive interactions of HT-29LMM cells with the pulmonary microvasculature showed comparable patterns, we used the same antibodies and protocols for integrin inhibition as described before [6,17] to determine the involvement of integrins in the metastatic cell arrest within the lung.

**Figure 4:**
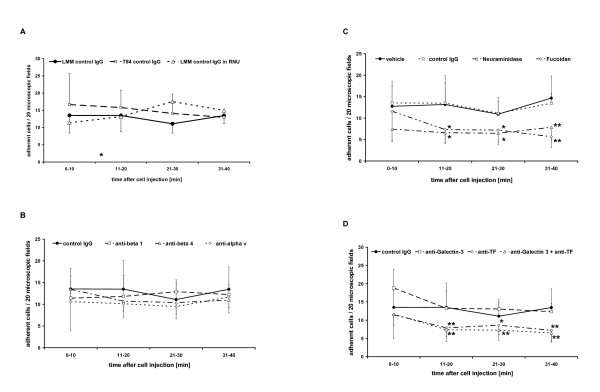
**Pulmonary tumor cell adhesion in vivo**. ***A: Adhesion of HT-29LMM and T84 colon cancer cells***. Determined by quantitative in vivo microscopy, human highly metastastic HT-29LMM and lung metastasis derived T84 colon cancer cells show similar adhesive properties in the rat lung. ***B: Integrin - inhibition ***Using quantitative in vivo microscopy of the lung, inhibition of colon cancer cells' integrins did not impair in vivo tumor cell adhesion in the pulmonary microvasculature. ***C: Selectin - inhibition ***Enzymatic hydrolisation of sialylated glycoprotein structures by Neumaminidase-V significantly impaired tumor cell adhesion within the pulmonary microvasculature. Furthermore, tumor cell arrest was impaired after treatment of the animals with fucoidan 15 minutes prior to cell injection for inhibition of P- and L-selectins. ***D: Thomson-Friedenreich antigen inhibition ***Tumor cell - endothelial cell adhesion in vivo was significantly reduced, but not completely lost, after inhibition of TF-antigen and galectin-3 at tumor cells.

After injection of HT-29LMM cells treated with unspecific mouse IgG (n = 9) 13.5 ± 5.1; 13.5 ± 4.8; 11.1 ± 2.7; and 13.5 ± 2.3 cells/20 microscopic fields were found adherent in the lung at 10; 20; 30; and 40 minutes after cell injection, respectively. These numbers were comparable to those from untreated cells. Neutralisation of various integrin subunits at the tumor cells' surfaces did not impair cell adhesion within the lung capillaries (Figure 4B; Table 1).

### Sialyl Lewis antigens can mediate adhesive tumor cell - microvascular endothelial cell interactions

After the identification of selectin binding sLe-a at the cells' surface and with respect to earlier reports indicating sLe-a mediated tumor cell adhesion within the liver [17], HT-29 LMM were pre-treated with neuraminidase type V for enzymatic hydrolization of sialylated residues as described before [17]. This treatment significantly impaired metastatic tumor cell adhesion in the lung as it did in the liver [17]. Using vehicle treated control cells 10.9 ± 3.9 to 15.0 ± 5.9 adherent cells/20 microscopic fields were counted in the respective time intervals. Enzymatic hydrolization of sialylated cell surface glycoproteins by incubation with neuraminidase-V significantly impaired early cell adhesion in vivo and the number of adherent cells was reduced: 6.5 ± 2.6 to 7.9 ± 3.0 cells/20 microscopic fields (p < 0.05 - 0.001). To confirm the involvement of selectins, animals were treated with fucoidan (0.8 mg/kg) 15 minutes prior to cell injection [19]. This treatment also significantly impaired adhesion of vehicle treated cells in the pulmonary microvasculature (Figure 4C; Table 1).

### Tumor cell adhesion is partly mediated by combined activity of Thomson-Friedenreich (TF) antigen and galectin-3

The TF antigen binding cell surface molecule Galectin-3 is likely involved in the adhesion of metastasizing tumor cells to endothelial cells [11,21]. We therefore treated control cells (n = 5) with unconditioned control medium prior to injection. At 10; 20; 30; and 40 minutes after cell injection 10.9 ± 5.1; 18.3 ± 10.1, 16.4 ± 3,9; and 14.8 ± 6.2 cells/20 microscopic fields were adherent within the pulmonary microcirculation. After incubation of the cells with unconditioned medium and addition of function blocking anti-galectin-3 antibodies (n = 5) prior to injection no significant differences in the numbers of adherent cells were observed. After incubation with JAA-hybridoma medium containing function blocking anti-TF mouse IgG, significantly reduced adhesion of HT-29LMM cells was observed. Simultaneous anti-galectin-3 and anti-TF treatment of HT-29LMM cells (n = 5) also resulted in a significant reduction of adherent cells within the lung (p < 0.05 - p < 0.001) (Figure 4D; Table 1), similar to anti-TF treatment alone.

## Discussion

The organ specificity of metastatatic TC arrest is not only determined by the repertoire of available adhesion molecules expressed at the cells' surfaces but in addition is influenced by the histological architecture, the availability, and organ specific exposure of possible adhesion sites within the targeted organ. In the case of colon cancer, the lung, next to liver and lymph nodes, is one of the most important metastasic targets. In this study, we demonstrate that metastatic cell arrest in the lung is exclusively initiated by adhesive interactions between circulating TC and the pulmonary microvascular endothelium. In contrast to the relevance of initial TC-ECM interactions for cell arrest within the hepatic sinusoids [6,17] direct matrix binding is not responsible for initial pulmonary tumor cell arrest. This points out the organ specific nature of metastatic tumor cell adhesion within potential target organs.

The lung's microvasculature is lined by a continuous endothelium, underlined by a basal membrane mainly composed of fibronectin, collagen IV and laminin. Anatomically defined fenestrae as documented for the kidneys' glomerula or the hepatic sinusoidal endothelium cannot be found in the pulmonary endothelium [25]. Although TC-EC adhesive interactions are of solely functional nature and earlier reports failed to observe morphologic correlations [26], the data presented herein support the role of TC - EC interactions in the initial phase of metastatic lung colonization. Adherent TCs leave a remaining perfused vessel lumen suggesting specific interactions rather than size restricted arrest. Furthermore, TCs passing the pulmonary capillaries were repeatedly observed in our study. Size restricted arrest, cannot be completely excluded by two dimensional in vivo microscopy, but 3D examination of in situ fixed and physiologically perfused lungs supports specific adhesion of colon cancer cells to the pulmonary capillary endothelium.

Despite the controversies about the potential role of size-restricted mechanical arrest for the initiation of organ colonization [4] a large body of data supports the role of specific adhesive interactions of circulating TCs within the capillary microenvironment of potential metastatic target organs [27]. This initial adhesion can be mediated by heterotypic TC - EC interactions and/or TC - ECM interactions [17,26,28]. Furthermore, the coagulatory system [29,30], including platelets [31] and possibly leucocytes [32] seems to interfere with metastatic tumor cell arrest.

Under physiological conditions, terminally fucosylated glycans such as sLe-x function as selectin ligands, e.g. on activated leucocytes [33], and mediate rolling on and adhesion to ECs [34]. But altered glycosylation of cell surface molecules is also a prominent feature of malignant cells [9,35]. These molecules appear to be critically involved in the organ tropism of metastasis formation. For example, sLe-a and sLe-x can mediate adhesion of differentiated epithelial cancer cells to endothelial E-selectin in vitro [36] and in vivo [28]. SLe-x was shown to initiate liver metastasis of human colon cancer cells [37] and high levels of sLe-x expression were correlated with poor survival of colo-rectal cancer patients [38]. E-selectin mediated adhesion triggers numerous functional alterations in adhering TCs [39] as well as in ECs. For example, adhering TCs induce further E-selectin expression in ECs [40], while adhering TCs are subjected to shear forces, inducing intracellular signalling like focal adhesion kinase phosphorylation and further adhesion stabilisation [41]. Confined disturbances in microvascular physiology may further be caused by local induction of nitric oxide after TC -EC interaction [42], as well as altered mechanical properties of TC by cytoskeletal rearrangement after cell adhesion [43]. Nevertheless, details of these biophysical alterations remain unclear.

In this study pre-treatment of the animals with fucoidan and pre-treatment of the injected cells with neuraminidase significantly impaired pulmonary TC arrest in vivo. Fucoidan mainly affects P-selectin and L-selectin with minor effects on E-selectin [44]. In contrast, neuraminidase inactivates sialylated adhesion molecules, such as sLe-a that is known to bind E- and P- selectin, and to a lesser extent also L-selectin. Nevertheless, adhesion of epithelial TCs to the microvascular endothelium may also be mediated by indirect cell adhesion, using additional cell types or soluble molecules within the blood [31,32]. Therefore, our results suggest the involvement of platelets and possibly leucocytes in in vivo adhesion of TCs in the lung. This is also supported by previous reports from other groups [31,32].

Direct heterotypic TC - EC adhesion seems to be also mediated by the TF and galectin-3 system [45]. The inhibition of tumor cell adhesion to EC by anti-TF treatment resulted in increased survival in a mouse model for spontaneous breast cancer metastases by impairing TC adhesion without affecting tumor cell proliferation [46]. Similar to earlier reports [9,11,17,32], we were able to detect a significant contribution of sLe-a - selectin mediated adhesion and TF - galectin-3 mediated TC adhesion within the lungs.

Beside TC - EC interactions, TC - ECM interactions, mediated by integrins were shown to be critically involved in the initiation of distant metastasis formation [12,47,48]. But in contrast to other typical metastatic target organs of colon cancer [6,10,17] we could not detect any significant contribution of ECM binding integrins to the initiation of pulmonary tumor cell adhesion. Although α3β1-integrin mediated TC adhesion to the basal membrane's laminin through single spot-like gaps of the endothelium was reported [49], we could not detect TC-ECM interaction in the early time frame analyzed.

The ECM components underlining the endothelium as the basal membrane are composed in an organ specific manner. Furthermore, the endothelial lining has specific characteristics in different organs that affect tumor cell adhesion. We recently reported that HT-29LMM passes the renal microcirculation without any signs of cell arrest [10]. On the other hand we found their organ specific adhesion within liver and lung [10]. In the kidneys' glomerula, the endothelium is discontinuously perforated by fenestrae, covering 20% of the endothelial surface [50] and the underlining ECM contains fibronectin beside collagen IV and laminin [51]. Inhibition of fibronectin binding α5β1-integrins on CHO cells critically impaired their adhesion within the kidney without affecting their adhesion in the lungs [52]. In contrast, CHO cells transfected with αvβ3-integrin exhibited enhanced accumulation in the liver by binding of extracellular vitronectin, while their accumulation in the lungs did not differ from their integrin αvβ3 negative parental cells [53]. In the liver the ECM is also directly accessible for circulating TCs through fenestrae of the EC covering 6-8% of the sinusoidal endothelial surface [54]. Fibronectin and type IV collagen in the space of Dissé were found mediating metastatic HT-29LMM cell arrest in the liver, while type I collagen predominantly seemed to mediate cell extravasation into the liver parenchyma [6,17,55]. In addition, TCs can directly adhere to small amounts of laminins via α6β1- and α6β4-integrins in the space of Dissé [17].

The in vivo microscopy technique used in this study provided quantitative information of TC arrest in the lung, but provided insufficient resolution to observe eventual morphologic changes of adhering TC or involved EC. Since the interface between capillary lumen and alveolar air is only 0.1 μm [25,54], flattening and transendothelial migration of tumor cells cannot be excluded, but extravasated tumor cells, e.g. cells in the alveolae, were not observed in formalin fixed tissue examined by confocal laser scanning microscopy. The fate of the adherent cells remains unclear in this study and may not be determined using the model and technique used here, but intravascular proliferation of adherent HT1080 sarcoma cells was described within the pulmonary capillaries by others [8]. In earlier studies, our group [10] and others [24] could not determine a correlation of cell adhesion and metastatic potential but found a positive correlation of metastatic potential and tumor cell extravasation of different colon cancer cells within the liver [10]. However, transendothelial migration that would be required within the lungs may take longer time periods since endothelial retraction and proteolysis of the pulmonary basal membrane are required in the lung, but only partially or not at all in the liver. As first adhesive interactions were observed as soon as 1 minute after cell injection and cell adhesion reached high numbers at 10 minutes after tumor cell injection, this seems not only to be a highly efficient process, but also a highly organ-specific step for the formation of metastatic lesions [24].

The continuous endothelium of the pulmonary capillary system appears to prevent direct integrin mediated adhesion of colon cancer cells to the underlining basal membrane. Although, single spots of exposed ECM can be found in the pulmonary capillaries [46], they do not provide sufficient binding capacity for integrin mediated adhesion. This is supported by the observation of TCs 'sticking' (temporary adhesion) without establishing firm adhesions, a phenomenon known for leucocytes but not documented for TCs in the liver [23]. As a result of initial TC-EC adhesion, conformational changes such as endothelial retraction and apoptosis of EC are well documented [56]. The subsequent exposure of underlining ECM components can provide additional adhesion capacity in a second step and may trigger further cell interactions. This could explain reports of increased lung metastasis of carcinoma cells after altered β1-integrin expression [57]. It would also be in line with reports of enhanced pulmonary metastasis of fibrosarcoma cells in mice after endothelial damage by bleomycin treatment that was associated with cells adhering to exposed basal membrane [58].

## Conclusions

In summary, this study is the first report using intravital in-situ observation of circulating colon cancer TCs within their important metastastatic target organ of the lung. Our results support the importance of organ specific histological architecture for metastatic TC colonization. While integrin mediated adhesion to ECM components may contribute to the initiation of metastatic tumor cell arrest in organs with a discontinuous endothelium like the liver, in organs with a continuous endothelial lining like the lung, initial metastatic TC adhesion appears to be exclusively mediated by direct and/or indirect TC - EC interactions via glycosylated adhesion molecules, such as selectins and TF-antigens.

## Abbreviations

TC: tumor cell; EC: endothelial cell; ECM: extracellular matrix; sLe-a: sialyl Lewis-a; sLe-x: sialyl Lewis-x; TF: Thomson-Friedenreich antigen;

## Competing interests

The authors declare that they have no competing interests.

## Authors' contributions

PG established the animal model, planned major parts of the study, contributed to analysing the data and drafted the manuscript. MK and STM generated the substantial body of the experimental data and contributed to the analysis of the results. JH gave the idea for the in vivo model and contributed to the interpretation of the data and drafting of the manuscript. All authors read and approved the final manuscript

## Pre-publication history

The pre-publication history for this paper can be accessed here:

http://www.biomedcentral.com/1471-2407/10/177/prepub
